# The position of entry point in total knee arthroplasty is associate with femoral bowing both in coronal and sagittal planes

**DOI:** 10.3389/fsurg.2022.935840

**Published:** 2022-07-18

**Authors:** Xianli Zeng, Yiming Yang, Zhenyu Jia, Jiarong Chen, Hongyuan Shen, Yan Jin, Yao Lu, Pingyue Li

**Affiliations:** ^1^ The First School of Clinical Medicine, Southern Medical University, Guangzhou, China; ^2^ Guangdong Key Lab of Orthopedic Technology and Implant Materials, Department of Orthopedics, General Hospital of Southern Theater Command of PLA, Guangzhou, China; ^3^ Department of Joint and Orthopedics, Orthopedic Center, Clinical Research Center, Zhujiang Hospital, Southern Medical University, Guangzhou, China

**Keywords:** femoral intramedullary guide, entry point, femoral bowing, intercondylar notch, total knee arthroplasty

## Abstract

**Objective:**

To investigate the femoral entry point of the intramedullary (IM) guiding rod applied to total knee arthroplasty (TKA) in Chinese subjects and the relationship with femoral bowing in the coronal and sagittal planes through three-dimensional (3D) validation methods.

**Methods:**

Computed tomography (CT) images of 80 femurs in Chinese subjects were imported into Mimics 19.0 to construct 3D models. All operations were conducted by Rhinoceros software 5.0. The position of the IM rod entry point was assessed by calculating the distance between the entry point and the apex of the intercondylar notch (AIN) in the coronal and sagittal planes. The coronal femoral bowing angle (cFBA) and sagittal femoral bowing angle (sFBA) were also measured.

**Results:**

The average optimal entry point was 0.17 mm medial and 12.37 mm anterior to the AIN in males, while it was 0.02 mm lateral and 16.13 mm anterior to the AIN in females. There was a significant difference between males and females in the sagittal plane (*t* *=* -6.570, *p *= 0.000). The mean cFBA was 1.68 ± 2.29°, and the mean sFBA was 12.66 ± 1.98°. The sFBA was strongly correlated with the anterior distance of the proper entry point, and the cFBA was moderately correlated with the lateral distance of the proper entry point.

**Conclusions:**

There was a strong correlation between the position of the entry point and the femoral bowing angle in both the coronal and sagittal planes. Thus, to achieve better alignment, the position of the entry point should be measured individually based on femoral bowing.

## Introduction

Total knee arthroplasty (TKA) is the most common surgical therapy for end-stage osteoarthritis of the knee, while proper implantation of the prostheses in TKA is essential for clinical outcome and patient satisfaction. Currently, several instruments have been applied to achieve proper alignment, including computer-assisted navigation, patient-specific guides, IM rods and robot assistance. Computer-assisted navigation can provide predictive information on the thickness of resection and the rotation angle and simulate lower extremity alignment after distinguishing several osseous points using a special instrument. However, errors can be found in the predictive osseous cutting level and implant size. Consequently, the sagittal alignment is still not as precise as coronal alignment (1). Patient-specific instrumentation is a technique based on preoperative CT/MRI data and 3D printing technology to create osteotomy guides without opening the medullary cavity, which theoretically improves the accuracy of osteotomy and reduces the operation time. However, many studies considered that patient-specific instrumentation did not show significantly more superiority than conventional instruments. There was no obvious advantage in improving alignment, reducing surgery time or decreasing the transfusion rate. Considering the expensive cost of patient-specific instrumentation, further research was performed to determine its disadvantages and advantages (2–4). The outcome of robot assistance used as an emerging technology has not been confirmed. Of all these methods, the IM rod is an economical and convenient instrument with reliable effects during TKA (5–7).

The classic entry point of the IM rod, most commonly adopted by surgeons, is 1 cm anterior to the femoral attachment of the posterior cruciate ligament, but there is a paucity of studies conducted to verify the accuracy. In addition, previous studies have found that potential errors in identifying the entry point could lead to the malalignment of the distal femoral cutting block by several degrees (8–12). Coronal malalignment contributes to higher surgery failure rates and lower quality of life (13, 14), while sagittal malalignment leads to patellar instability, maltracking and anterior knee pain (15, 16). Thus, it is important to identify the proper entry point when using an IM rod.

In the case of applying the same entry point, it was hypothesized that femoral bowing impacted the depth and orientation of the IM rod, which eventually influenced postoperative alignment. However, the relationship between the position of the entry point and femoral bowing has not yet been investigated. Therefore, the purpose of our research was to explore the proper entry point and evaluate the relationship between the position of the entry point and the femoral bowing angle.

## Methods

### Demographic data

From January 2019 to December 2020, 80 Chinese patients (mean age 42.44 ± 17.59, range 18–86 years) were recruited in our study. According to the order of admission, patients who met the following criteria were included after confirmation by imaging examination: (i) patients with intact cognitive function over the age of 18 (no sex restriction); (ii) patients diagnosed with knee osteoarthritis (Kellgren-Lawrence ≤2) before surgery; (iii) patients diagnosed with isolated or combined ligament injury of the knee joint; and (iv) patients diagnosed with meniscus injury regardless of the degree. Among them, the first condition had to be satisfied. Patients who met one of the following criteria were excluded: (i) patients with a previous history of lower extremity surgery; (ii) patients with lower limb muscle dysplasia; (iii) patients with obvious deformities of the lower limb; (iv) patients with obvious osseous defects; and (v) patients with any other skeletal disorders, such as bone tumors.

Recruitment was stopped when the number of both males and females was 40. The mean body mass index (BMI) was 29.9 ± 3.6 kg/m^2^ (range 16.65–31.63 kg/m^2^). In terms of age and BMI, there was no statistically significant difference between males and females (Table 1).

**Table 1:** Demographic data of participants.

	Male	Female	Total
Number	40	40	80
Lateral meniscus tear	7	5	12
Medial meniscus tear	5	4	9
ACL tear	11	2	13
PCL tear	3	0	3
ACL + Lateral meniscus tear	6	2	8
ACL + Medial meniscus tear	3	2	5
ACL + MCL tear	3	1	4
Knee osteoarthritis	2	24	26
Age, y, mean ± SD	42.80 ± 18.27	42.08 ± 17.11	42.44 ± 17.59
BMI, kg/m^2^, mean ± SD	24.43 ± 2.44	23.54 ± 3.60	23.99 ± 3.08

*ACL, anterior cruciate ligament; PCL, posterior cruciate ligament; MCL medial collateral ligament; BMI, body mass index.*

Our institutional review board approved the project. All patients signed informed consent and underwent lower extremity CT scanning (SOMATOM Emotion 16, Siemens Healthcare Gmbh, Germany; slice thickness = 0.625 mm). The radiography images were saved in Digital Imaging and Communications in Medicine (DICOM) format, which was then converted into a Mimics file to construct a 3D femur, which, in turn, was imported into Rhinoceros software 5.0 (Robert McNeel & Associates, USA) to perform the measurements.

### Coordinate system

**Figure 1:**
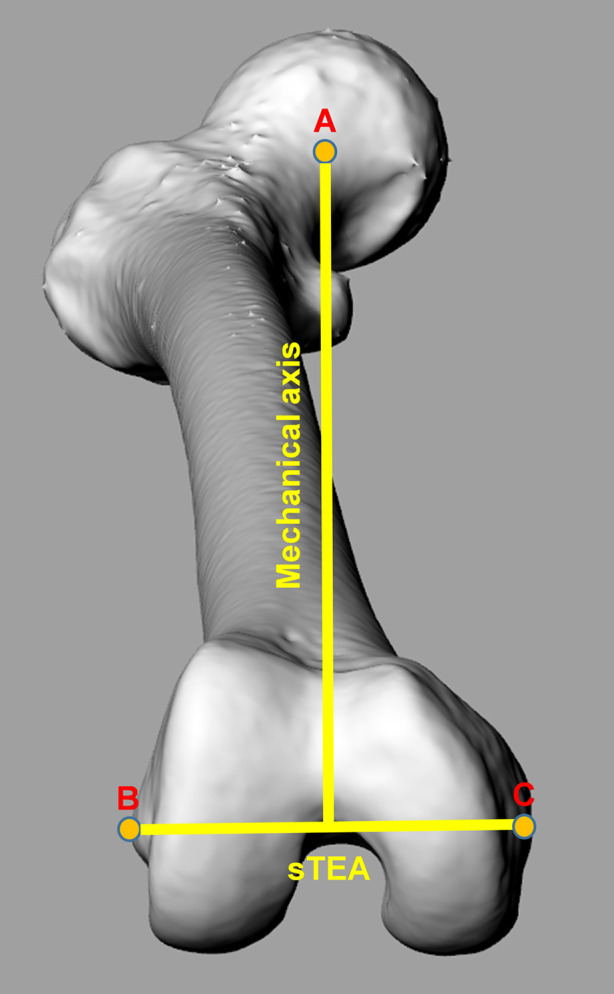
Coordinate system of femur. The surgical transepicondylar axis (sTEA) connecting the most convex point of the lateral condyle and the most concave point of the medial condyle was defined as the X-axis. The mechanical axis (MA) running from the geometric center of the femoral head to the midway point of the sTEA was regarded as the Y-axis. Consequently, the Z-axis was perpendicular to the plane established by the X-axis and Y-axis. Point A represents the geometric center of the femoral head. Point B represents the most convex point of the lateral condyle. Point C represents the most concave point of the medial condyle.

The surgical transepicondylar axis (sTEA) connecting the most convex point of the lateral condyle and the most concave point of the medial condyle was defined as the X-axis. The femoral mechanical axis (MA) running from the geometric center of the femoral head to the midway of the sTEA was regarded as the Y-axis. Consequently, the Z-axis was perpendicular to the plane established by the X-axis and Y-axis (Figure 1).

### Measurements

**Figure 2:**
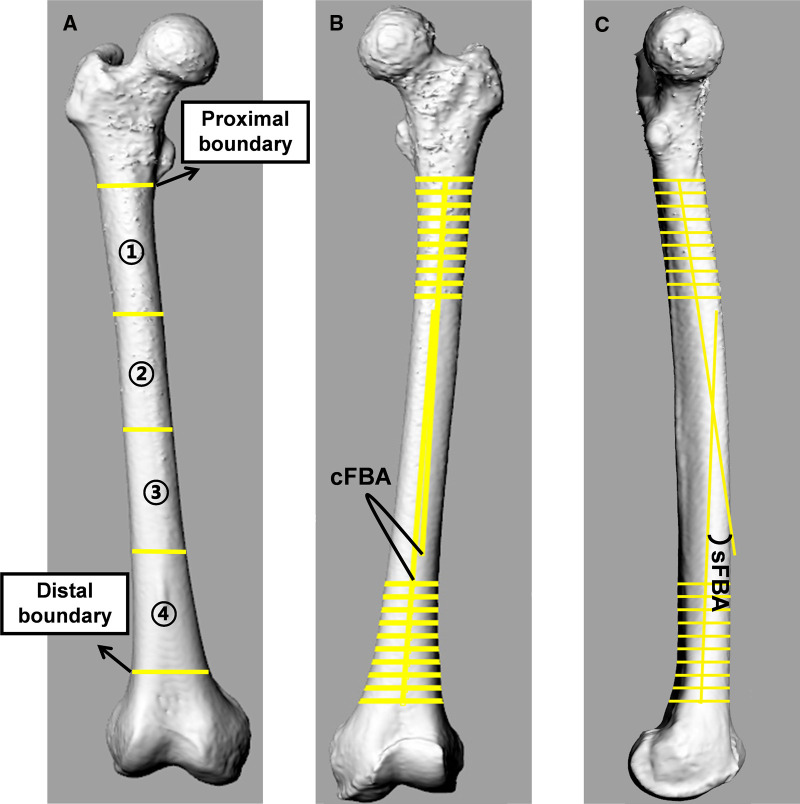
Femoral bowing in coronal and sagittal planes. (**A**) The femoral shaft was divided into four equal parts in the space. The proximal border was the lower edge of the lesser trochanter, and the distal border was the juncture between the femoral shaft and condylar region. (**B,C**) The angle between two midlines fitted by the points of 10 isometric cross-sections of proximal and distal femoral shaft sections was defined as the coronal femoral bowing angle (cFBA) in the coronal plane and the sagittal femoral bowing angle (sFBA) in the sagittal plane.

The femoral shaft was separated into four equal sections in space. The proximal border was the lower edge of the lesser trochanter, and the distal border was the juncture between the femoral shaft and condylar region. The angle between two midlines fitted by the points of 10 isometric cross-sections of proximal and distal femoral shaft sections was defined as the cFBA in the coronal plane and the sFBA in the sagittal plane (Figure 2). A positive cFBA value represented lateral femoral bowing, and a negative value represented medial femoral bowing. There was no negative value for the sFBA.

**Figure 3:**
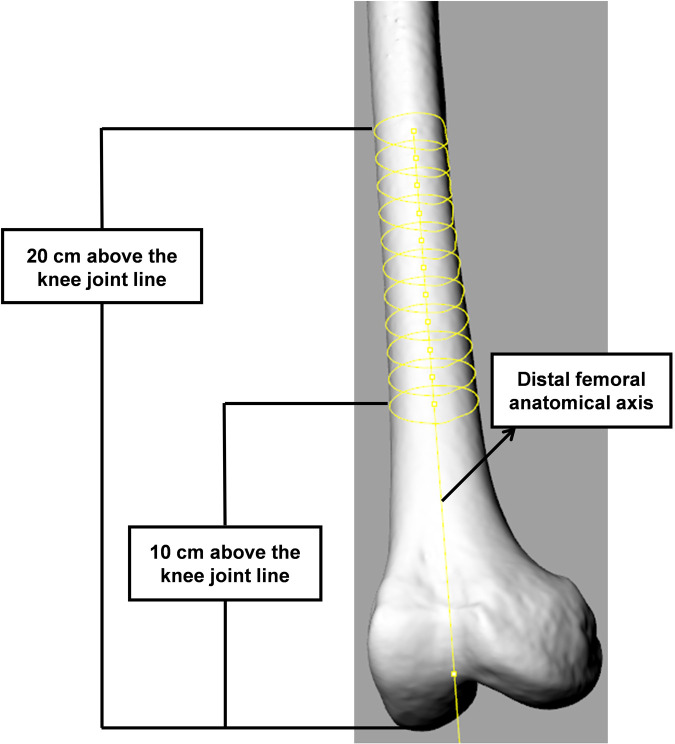
Identification of proper entry point. The anatomical axis was defined as the line fitted by the points of 10 isometric cross-sections of the femoral shaft at 10 and 20 cm above the distal femoral articular surface.

**Figure 4:**
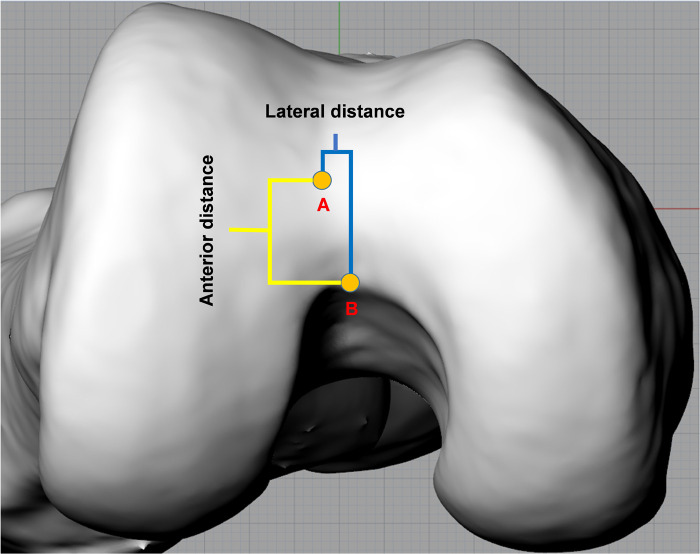
Measurement on axial plane. The anterior distance (highlighted by yellow line) represents the distance between the apex of the intercondylar notch (AIN) and entry point in the sagittal plane. The lateral distance (highlighted by blue line) represents the distance between the AIN and entry point in the coronal plane. Point A and B represent the AIN and entry point, respectively.

The proper entry point was determined as the point at which the anatomical axis of the femur intersected the distal femoral articular surface (Figure 3). The AIN was used as a reference point to determine the position of the proper entry point. The distance between the AIN and entry point was measured in the coronal and sagittal planes (Figure 4). The value was positive if the entry point was medial to the AIN, and it was negative when the entry point was lateral to the AIN in the coronal plane. In the sagittal plane, there was no negative value because all entry points were anterior to the AIN.

Three weeks after the initial measurement, 20 participants (10 females and 10 males) were randomly selected, and all parameters were measured a second time by the same observer and a different observer to evaluate intra- and interobserver reliability.

### Statistical analyses

The data were analyzed in SPSS 25.0 (IBM Crop. Armonk, NY, USA) and PASS 15.0 (NCSS, LLC. Kaysville, Utah, USA). A *p* value <0.05 was considered statistically significant for all analyses. The parameters between males and females were analyzed by independent t tests. The relationship between the distance and angle of all subjects was analyzed by Pearson's correlation coefficients. A *post hoc* power analysis was used to evaluate whether the sample size was sufficient to identify the difference between males and females.

## Results

**Figure 5:**
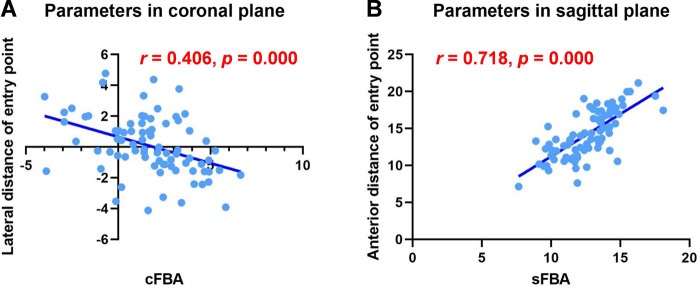
Linear regression of parameters in coronal and sagittal planes. (**A**) The sFBA showed a strong correlation with the anterior distance of the proper entry point in the sagittal plane (*r *= 0.718, *p *= 0.000). (**B**) The cFBA showed a moderate correlation with the lateral distance of the proper entry point in the coronal plane (*r *= 0.406, *p *= 0.000).

The average values of the entry point were 0.17 ± 1.86 mm (range, −4.13–4.77 mm) medial and 12.37 ± 2.39 mm (range, 7.14–18.96 mm) anterior to the AIN in males. The average values of the entry point were 0.02 ± 2.00 mm (range, −3.91–4.38 mm) medial and 16.13 ± 2.72 mm (range, 9.94–21.15 mm) anterior to the AIN in females. There was a significant difference between males and females in the sagittal plane (*t* *=* -6.570, *p *= 0.000). The *post hoc* power analysis showed that the actual power was 0.92 (>0.80), indicating that significant differences between males and females could be identified. However, there was no significant difference between males and females in the coronal plane (*t = *0.436, *p *= 0.664). The mean cFBA was 1.68 ± 2.29° (range, −3.99–6.64°), and the mean sFBA was 12.66 ± 1.98° (range, 7.66–18.11°) in all subjects (Table 2). The sFBA showed a strong correlation with the anterior distance of the proper entry point in the sagittal plane (*r *= 0.718, *p *= 0.000), and the cFBA showed a moderate correlation with the lateral distance of the proper entry point in the coronal plane (*r *= 0.406, *p *= 0.000). For every one-degree increase in the sFBA, the approach shifted anteriorly by an average of 1.15 mm (Figure 5). The intraclass correlation coefficient (ICC) of intraobserver reliability among all data sets was greater than 0.80 (Table 3), and the ICC of interobserver reliability among all data sets was greater than 0.80 (Table 4). According to their relationship, a mathematical predictive model was established as follow: Lateral distance = −0.341 × cFBA + 0.652, Anterior distance = 1.148 × sFBA - 0.281.

**Table 2:** Data analysis comparison between males and females.

	Medial to the AIN	Anterior to the AIN	cFBA	sFBA
Male	0.17 ± 1.86	12.37 ± 2.39	1.13 ± 2.43°	11.89 ± 1.66°
Female	0.02 ± 2.00	16.13 ± 2.72	2.24 ± 2.03°	13.42 ± 2.00°
*t*-value	0.436	−6.570	−2.208	−3.697
*p*-value	0.664	0.000	0.030	0.000
Total	0.08 ± 1.92	14.25 ± 3.17	1.68 ± 2.29°	12.66 ± 1.98°

*AIN, apex of the intercondylar notch; cFBA, coronal femoral bowing angle; sFBA, sagittal femoral bowing angle.*

**Table 3:** ICC of intraobserver reliability.

	The first observer	The same observer	ICC	*p*-value
Medial to the AIN	0.42 ± 1.74	0.07 ± 1.41	0.927	0.000
Anterior to the AIN	13.69 ± 2.97	13.43 ± 3.04	0.993	0.000
cFBA	0.41 ± 2.48°	0.46 ± 2.53°	0.987	0.000
sFBA	12.73 ± 1.52°	12.51 ± 1.71°	0.942	0.000

*ICC > 0.80, p < 0.000 indicated great consistency between two groups.*

**Table 4:** ICC of interobserver reliability.

	The first observer	The second observer	ICC	*p*-value
Medial to the AIN	0.42 ± 1.74	0.03 ± 1.46	0.906	0.000
Anterior to the AIN	13.69 ± 2.97	13.43 ± 2.91	0.988	0.000
cFBA	0.41 ± 2.48	0.53 ± 2.58	0.977	0.000
sFBA	12.73 ± 1.52	12.31 ± 1.87	0.890	0.000

*ICC > 0.80, p < 0.000 indicated great consistency between two groups.*

## Discussion

In our study, the anterior distance of the entry point had strong positive correlations with the sFBA, and the lateral distance of the entry point had a moderate positive correlation with the cFBA. This study is the first to investigate the association between the entry point and femoral bowing in the sagittal plane utilizing a 3D validation approach. Additionally, the current study showed that the proper entry point of the female femur was located more anterior to that of the male femur in the sagittal plane.

In general, the determination of the entry point is the first step in performing distal femoral osteotomy after the removal of osteophytes. Previous studies have demonstrated that IM guides lead to better outcomes. HAL et al. found that the IM femoral alignment system had more accuracy than the extramedullary femoral alignment system after comparing a series of angles through roentgenographs after TKA (17). Antonio et al. also considered that intramedullary guides had better coronal alignment after conducting a radiographic analysis according to the postoperative alignment angle (5). We could not determine the sway of the rod within the marrow cavity of the femur. Consequently, errors could be found when performing the distal osteotomy of the femur. As far as we know, the position of the entry point, IM rod length, diameter, orientation and distal femoral cut angle influence both coronal and sagittal alignment (9). Many studies on potential angle errors made by the orientation of IM rods have been performed. Usually, a safe boundary of 3° is recommended to achieve neutral mechanical alignment, which can optimize implant durability (18–21). However, Jianlin et al. found that the potential angle error was 2.68° at most in the coronal plane and 3.68° at most in the sagittal plane when taking the intercondylar notch as the entry point (18–21). J. Novotny et al. found that the maximum mean potential error caused by the sway of the rod within the marrow cavity of the femur was 5.78° in the coronal plane and 5.02° in the sagittal plane (11).

However, identifying a proper entry point might decrease the potential angle error caused by the IM rod. Yohei et al. determined that the mediolateral deviation was 1.4° at maximum and the anteroposterior deviation was 1.6° when adopting an entry point similar to ours (12). Through an analogous method, Xiao et al. calculated that the possible deviation in the coronal plane was 0.88 ± 0.21° and 1.13 ± 0.39° in the sagittal plane (8). Ma et al. also believed that taking the position where the femoral anatomical axis intersected the distal femoral articular surface rather than the femoral trochlear groove or the point 10 mm anterior to the femoral attachment of the posterior cruciate ligament would greatly reduce the potential inaccuracy (10). All of these previous studies proved that the method we used to identify the entry point was dependable.

In a study conducted by Yongsak et al., the correct entrance site was discovered to be 1.5 ± 2.01 mm medial and 12 ± 2.72 mm superior to the AIN (22), which was similar to our results. However, their measurements depended on anterior-posterior and lateral X-radiography. According to Xiao et al., the entry point was 2.94 ± 1.12 mm (range, 0.79–4.91 mm) medial and 6.01 ± 2.09 mm (range, 2.49–9.51 mm) anterior to the AIN (8), which was quite different from our results. Possibly, we recruited patients with a wider age range (mean age 42.44 ± 17.59, range 18–86 years vs. 25.6 ± 2.9, range 18–29 years). Ma et al. determined that males had an entry point that was 1.49 ± 0.92 mm (range, 0.32–3.76 mm) medial and 13.39 ± 2.46 mm (range, 9.36–17.60 mm) superior to the AIN, whereas females had an entry point that was 1.77 ± 1.04 mm (range, 0.24–4.45 mm) medial and 15.29 ± 3.44 mm (range, 9.21–21.65 mm) superior to the AIN (10). Their study, like ours, involved patients of the same race and the same method of identifying the entry point. The measurements that they found were also close to ours. However, they concentrated on investigating the accuracy of the method, while we focused on the correlation between the femoral entry point and femoral bowing.

Previous studies on the influence of sagittal distal femoral bowing on TKA have been conducted. Tameem et al. found that men had less distal bowing than women (23). Another study concluded that a sex difference found in the sagittal bowing of the distal femur showed that females had a larger angle of sagittal bowing than males, which eventually affected the sagittal position of the femoral component (24). This difference may be why the entry point of the female femur was located more anterior to that of the male femur in the sagittal plane. However, neither of them proposed a practical and feasible approach to handle femoral bowing during surgery. Rajshekhar et al. also considered that every one-degree increase in lateral femoral bowing lateralized the position of the entry point by an average of 1.04 mm after performing a series of TKAs by selecting the lateralization of the femoral entry point (25). They focused only on patients with lateral femoral bowing more than 4°, who made up just 16% of the participants enrolled in our study. It has been reported that the percentage of patients with lateral bowing with >2° angulation among patients with knee osteoarthritis was just 44% (26), which suggested that there were few patients with lateral femoral bowing greater than 4°. Perhaps this was the reason why they observed a strong correlation between the lateral distance of the proper entry point and the cFBA (R^2^ = 0.67, *p *= 0.00), which was moderately correlated in our study. In addition, they evaluated the cFBA by preoperative and postoperative radiographs, which might neglect the rotational alignment of the lower extremities. The same method to measure the cFBA was used in both their study and ours. However, we used a 3D model. Yau et al. found that the cFBA was 1.6 ± 4.4° through a similar measurement on radiographs (26), which was supported by the current study.

The current study had several limitations. First, the number of participants in this study was limited and we did not take advanced osteoarthritis patient into consideration for morphologic changes on femoral condyle. Second, we did not take other factors, such as the radius of the entry point and the length of the femur, into account, which might affect the results. Third, the morphology of femur medullar cavity will influence the depth and orientation of IM rod and measurement of medullar cavity bowing angle will be useful in clinic. Unfortunately, to the best of our knowledge, there are still no widely recognized and practical method to measure the medullar cavity bowing angle involved in 3D reconstruction of bone cortex. Furthermore, the measurement was based on 3D model, which might be difficult to be applied to routine procedures. Finally, future study about patient outcomes would be needed to verify the effectiveness for an individual entry point.

## Conclusion

There was a strong correlation between the position of the entry point and the femoral bowing angle in both the coronal and sagittal planes. Surgeons could determine the position of the entry point based on coronal and sagittal femoral bowing to achieve better alignment in the coronal and sagittal planes.

## Data Availability

The original contributions presented in the study are included in the article/Suplementary Material, further inquiries can be directed to the corresponding author/s.
